# Condition and Phenotype-Dependent Dispersal in a Damselfly, *Calopteryx splendens*

**DOI:** 10.1371/journal.pone.0010694

**Published:** 2010-05-18

**Authors:** Audrey Chaput-Bardy, Arnaud Grégoire, Michel Baguette, Alain Pagano, Jean Secondi

**Affiliations:** 1 Laboratoire d'Etudes Environnementales des Systèmes Anthropisés, Université d'Angers, Angers, France; 2 Centre d'Ecologie Fonctionnelle et Evolutive, UMR 5175, Montpellier, France; 3 Equipe EvolTrait, UMR 7179 CNRS-MNHN, Brunoy, France; 4 CNRS USR 2936 Station Expérimentale du CNRS à Moulis, Moulis, France; University of Alabama, United States of America

## Abstract

Individual dispersal decisions may be affected by the internal state of the individual and the external information of its current environment. Here we estimated the influence of dispersal on survival and investigated if individual phenotype (sex and wing length) and environmental condition (conspecific density and sex-ratio) affected dispersal decisions in the banded damselfly, *Calopteryx splendens*. As suspected from the literature, we showed that the proportion of dispersing individuals was higher in females than in males. We also found negative-density dependent dispersal in both sexes and influence of sex-ratio on dispersal. Individuals moved less when sex-ratio was male biased. These results are consistent with a lek mating system where males aggregate in a place and hold mating territories. Contrary to our expectations, neither dispersal nor survival was affected by wing length. Nevertheless, mean adult survival was about 8% lower in dispersing individuals than in residents. This might reflect a mortality cost due to dispersal.

## Introduction

Dispersal is central to many ecological and evolutionary processes, from metapopulation dynamics to metacommunity evolution, through gene flow [1], [2], . Limited effective dispersal (gene flow) under various environmental conditions may increase genetic differentiation of populations that ultimately might lead to speciation. Dispersal has long been considered as a fixed species-specific process. However, it is now more widely accepted that dispersal can be both condition and phenotype-dependent [3], [6], [7]. Many debates in ecology arise through semantic differences [2]. There is clearly a semantic issue about condition dependent dispersal in the current dispersal theory. “Condition” referred initially to the internal state of the individual, whereas “context” referred to the environmental conditions (e.g. [1]). However, to unify dispersal theory with ecological modelling practices, “condition” refers now to the environmental conditions acting on the individual [7]. Accordingly, “context dependent dispersal” has disappeared from the current dispersal theory, whereas “phenotypic dependent dispersal” means now dispersal depending on the internal state of the individual, which is affected by many environmental factors like starvation, parasite load, density or sex-ratio. Because individual phenotype (e.g. sex or body weight) and environmental conditions often have strong effects on fitness, these factors are considered as the main drivers of dispersal ecology and evolution in animals. However, how these factors interact remains poorly understood (e.g. [4]).

Insects provide good biological models to investigate how condition and phenotype affect dispersal. Dispersal in insects usually depends on morphological traits involved in flight, like wing length or thorax muscle mass [8], [9], [10], [11]. Within a species long-winged individuals commonly disperse over longer distances than short-winged ones [12], [13], [14]. In addition to the genetic control of wing development reported in some species [15], environmental factors can directly affect wing development and the flight apparatus [16]. Food quality experienced during larval stages often influences the proportion of dispersers or dispersing morphs [17]. If individuals are limited in their “choice” of an environment in which to develop (e.g. damselfly larvae are limited to where oviposition occurs), then food quality can affect two “levels” of dispersal: i) adult dispersal to find better quality habitats in which to mate/oviposit, ii) poor quality habitats limiting the dispersal of adults who develop as larvae in that habitat, i.e. by virtue of the negative effects the habitat had on wing development. High conspecific density also promotes dispersal [18], [19] as it tends to increase competition for resources [20], or sexual harassment [21]. Positive density-dependent dispersal is common in insects such as plant-hoppers [22], [23], beetles [24], butterflies [25], [26] and flies [27]. However, a negative relationship between density and dispersal has sometimes been reported [21], [28], [29], [30]. This relation might be explained by the fact that high conspecific density acts as a signal of good quality habitats or of increased mating opportunities.

These contrasting results demonstrate that the relationship between density and dispersal is complex [31]. Positive density-dependent dispersal (dispersal increasing with density) is expected when density is perceived as a proxy for competition intensity, often when there is strong spatial autocorrelation in environmental stochasticity (i.e. when environmental conditions are correlated across space, and hence are predictable). Negative density-dependent dispersal (dispersal decreasing with density) is in turn expected when density is perceived as proxy for habitat quality, often when there is weak spatial autocorrelation in environmental stochasticity (i.e. environmental conditions are not predictable from the spatial context [17]). Individual phenotype and population density are not necessarily independent. They may interact to determine individual fitness component such as survival. For instance, survival can correlate with body size, i.e. selection favouring larger individuals [32], [33], [34], and can be affected by density and/or a high proportion of males (male biased sex-ratio), i.e. increasing costs of male territorial behaviour and male-male aggression at high density decrease survivorship [35], [36].

Here we investigated how phenotype, i.e. sex and wing length, and condition, i.e. population density and sex-ratio, influenced dispersal behaviour and survival in the damselfly *Calopteryx splendens*, Harris 1782 (Odonata: Zygoptera). Male *C. splendens* are supposed to be territorial [37], [38]. Whether they defend a single place during their adult life, and whether territories are stable or moving is not really known. Females patrol in areas of high male density. As in other calopterid species, the mating system seems therefore to correspond to a lek [39]. From the literature and our field experience, the majority of individuals establish home ranges that cover ca. 50 m along the river [40], [41]. We decided accordingly to use sections of 50 m length as basic units for the definition of dispersal. This means that we defined here dispersal as *movements leaving individuals outside of their home ranges*, which is a commonly used definition for model species where dispersal depends more on social factors than on environmental conditions (i.e. the common lizard [7]). Given the lek mating system of *C. spendens*, we indeed anticipated that social interactions played a crucial role in the determinism of dispersal in this species. Dispersal is usually considered as a three step process: emigration, transfer and immigration [7], [42], [43], [44]. We focused here only on the first step of dispersal, i.e. the decision to leave a suitable habitat.

Based on classical assumptions that large body size is favoured in territorial males [45],[46], we predicted that larger individuals control more frequently mating territories and consequently disperse less from their section of origin than smaller individuals. An alternative hypothesis is that larger males are able to disperse farther before finding a suitable mating place. Because of male harassment and differential habitat use (females forage and wander further than males away from their natal streams), a female biased-dispersal is expected in calopterids but this has not been shown yet (see [47]). For density, opposing predictions can be made as well depending on whether intraspecific competition for resources or attractiveness of good quality breeding sites prevails (see above). According to a study on another damselfly [30], [48] and the gregarious tendency of *C. splendens*, a negative relationship between density and dispersal was expected. We also investigated the relationships between survival and dispersal. A simple prediction is that dispersal is costly, dispersers having a lower survival rate or fecundity [49], [50], [51], [52] than residents respectively due to predation risk [53], [54] or energetic cost [49], [55], [56] for instance. However, explaining a negative relationship between dispersal and survival is not straightforward. It could be caused by indirect effects, for instance if survival depends on density or wing length. We thus tried to disentangle the direct and indirect effects of these variables on dispersal.

## Materials and Methods

### Study species and basic field methods

*Calopteryx splendens* is a damselfly that emerges between late April and early September [57]. The maturation stage lasts several days and adults live 3 to 6 weeks. Mating and oviposition take place exclusively at or near the water surface [38]. Adult males are known to be territorial and sedentary. Individuals usually patrol on zones of about 50-m length along the watercourse (e.g. stream), where males establish temporary territories by defending one perching site and its immediate surroundings [40], [41].

Capture sessions and surveys were carried out during the peak of the breeding season from 17^th^ of June to 20^th^ of July 2006 in the Loir stream (Briollay, France, 47°33′22.3″N, 0°31′31.3″W). In order to limit individual disturbances, we sampled three successive days every three days except during bad weather conditions when mature adults were inactive. We carried out one transect longitudinal to the watercourse. The transect was 1050-m long and was divided in 10-m long sections by colour marks attached to the vegetation. In previous studies, authors reported that more than 80% adult *C. splendens* moved less than 100 m from their initial capture site [40], [41]. We thus selected transect length to minimize the effect of individuals dispersing out of transect during the study, as such individuals could bias dispersal estimates. A 10-h capture-mark-recapture (CMR) operation was conducted on each capture session day. We alternated the starting point of transect between surveys of the same site so that half of surveys started from the upstream subsections, and half from downstream subsections. Each 10-m subsection was searched for unmarked and marked individuals by walking along the stream. Individuals of both sexes were captured using a standard insect net and individually marked by a combination of a colour code written on wings with permanent ink. We captured already marked individuals to identify them. The position of captured individuals within a subsection was estimated visually so that each individual could be located to the nearest meter. Individuals were released in the middle of the subsection in which they were captured. Only mature individuals were caught. The length of the left forewing (from the base to the tip of the wing) was measured with a digital calliper to the nearest 0.01 mm. Wing length is commonly used as a non-invasive index of body size [38]. Furthermore, wing length is positively correlated with wing width, thorax length, thorax width, thorax mass, and femur length [58]. Due to time constraints (mean handling time of five minutes per individual), we measured only a sub-sample of the total number of marked individuals (150/1159). Basic statistics (i.e. median distance move, mean sex-ratio, and mean density) were computed using the free software R (http://www.r-project.org/).

### Capture-mark-recapture multistate models

For CMR analyses the 1050-m transect was divided into 50-m long sections as most of individuals moved less than 50 m. Survival probabilities between two consecutive capture sessions were estimated through CMR statistical modelling. Here we present the calculation of survival for unisite models in order to understand the estimation of this parameter. Survival *S* is the probability of surviving from occasion *i* to *i*+1 and *p* is the probability that if alive and in the sample at time *i*, that the individual will be encountered. So, *S*_1_ is the probability that an animal encountered and released alive at sampling occasion 1 will survive the interval from occasion 1 to occasion 2, and so on. Similarly, *p*_2_ is the probability that conditional on the individual being alive and in the sample, that it will be encountered at occasion 2, and so on. Now, if we encounter the animal, we record it in our data as ‘1’ (the animal was seen). If we do not see the animal, it is a ‘0’. So, based on a 3 days study, an animal with an encounter history of ‘111’ was seen in the first day (the marking day), seen again in the second day, and also seen in the third day. Compare this with an animal with an encounter history of ‘101’. This animal was seen in the first day, when it was marked, not seen in the second day, but seen again in the third day. For instance, of the 30 individual marked and released alive, 5 were encountered on both sampling occasion 2 and sampling occasion 3 (encounter history of 111), 10 were encountered on sampling occasion 2, but were not seen on sampling occasion 3 (encounter history of 110), 2 were encountered on sampling occasion 3 only (encounter history of 101), and 13 were not recaptured (encounter history of 100). As noted by [59], because animals with the same recapture history have the same probability expression, then the number of individuals observed with each encounter history appears as an exponent of the corresponding probability in the likelihood. Thus, it is written:
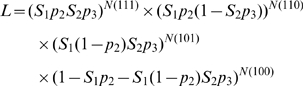
where *N*_(ijk)_ is the observed frequency of individuals with encounter history *ijk*.

As with the binomial, the log transform of the likelihood expression is taken, and after substituting the frequencies of each capture history, the following equation is obtained:


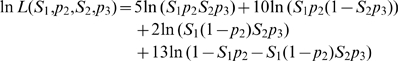
 [60].

The program MARK [60] derives the estimates of the parameter *S* and *p* that maximize this likelihood.

Unisite mark-recapture models cannot distinguish between survival and emigration [59] which may lead to biased estimates of survival if emigration is related to phenotypic trait values (e.g. [61]). Monitoring individuals in multiple sites (so-called multistate MR models [62]) can solve this problem by estimating phenotype-dependent movement among sites [63]. Here we used such a multistate MR model that takes into account the spatial location of each individual at each capture occasion. According to the daily pattern of capture sessions, a capture history was constructed for each individual, which consisted of a series of 1 (when an individual was first captured on its 50-m section or recaptured on the same section), 0 (when an individual was not captured) and 2 (if an individual was recaptured out of its 50-m section). Thus, individuals that left their 50-m section of origin were considered as dispersers (state 2) and individuals that stayed in the section as residents (state 1). Density and sex-ratio estimates in each section at each capture session were inferred from the number of individuals in 50-m sections on each capture session. Moreover, our linear study system was flanked on both sides by two large areas (700 m length) of unsuitable habitats. Given the low dispersal ability of the species [40], [64], this setting should minimize the risk of emigration out of the study system.

We first tested the effect of time (t = day), state (s1 = resident and s2 = disperser), and sex (i.e. male or female) on survival (S), recapture probability (p), and the emigration probability (psi = transition probability from the section of origin to another, i.e. disperse or not) using MARK [60]. MARK makes possible to take into account the interval between different capture periods. Hence, time lags of several days between two consecutive capture sessions were accounted for in the models.

The validity of estimates obtained from survival models requires that several assumptions are met [59]. The absence of structural problems in the dataset and the assumption that animals behave independently (e.g. capture does not affect recapture probability) must be verified. These assumptions are usually tested using the Cormack-Jolly-Seber (CJS) model that assumes survival (S) and recapture probability (p) to be dependent on time (t). The test of goodness of fit on multistate model was performed with U-CARE [65]. This model offered a satisfactory fit to the dataset (Table 1). Thus, there were no significant departures from assumptions, allowing the use of CMR statistics. We started from model {S(state*sex*t)p(state*sex*t)psi(state*sex*t)} which included an interaction term between state according to dispersal, sex, and time (t) on survival (S), recapture probability (p), and emigration probability (psi) to model {S(.)p(.)psi(.)} where survival, recapture probability, and emigration probability remained constant. As we had no a priori expectation about which variables influence each factor (i.e. model structure), we tested all models starting by simplifying recapture probability, then survival probability, and finally emigration probability. We performed a second model selection using the most parsimonious model from the precedent section. At this stage, we included three covariates (density, sex-ratio, and wing length) on the demographic parameters when it was biologically meaningful. Thus density, sex-ratio (#males/#females), and wing length were considered for survival and emigration probabilities, only wing length was considered for recapture probability because individuals' phenotype instead of environmental conditions is supposed to affect recapture probability of individuals. Density and sex-ratio were respectively log and arcsin square root transformed to reach normality. Competing models were compared by means of the corrected Akaike's Information Criterion, AICc [66]. Likelihood Ratio Tests (LRT), were performed to test the significance of specific effects when competing models had AICc lower than 2.

**Table 1:** Results of goodness-of-fit tests of the general multistate Capture-Mark-Recapture Model.

Test component	Chi2	d.f.	p value
Males			
TEST 3G	28.074	45	0.977
TEST M	9.330	14	0.809
GOF Test for the JMV Model	37.404	59	0.987
Females			
TEST 3G	3.789	19	0.990
TEST M	7.012	5	0.220
GOF Test for the JMV Model	10.801	24	0.990

**JMV Model**: ‘Jolly Move’ model, this GOF test is based on the property that all animals present at any given time behave in the same way.

**Test 3G** assumes ‘behavioural equivalence’ of individuals released together regardless of their past capture history.

**Test M**, which tests ‘equivalence’ among those individuals that are eventually recaptured (on a subsequent occasion) conditional on whether or not they are encountered at the present occasion.

## Results

**Figure 1:**
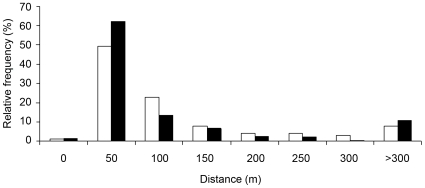
Distribution of distance covered by captured individuals for males (in black) and females (in white).

A total of 655 males and 504 females were caught. Within 50-m section, male-to-female sex-ratio was 1.30±0.33 (mean ± SD) and density was 88.41±48.17 individuals/section. Recapture rate was 45% for males and 21% for females. Most males (77%) and females (73%) were recaptured less than 150 m from their initial capture site and only 7% of males and 3% of females moved over 500 m during the study (Fig. 1). The maximal distance covered by a male was 865 m and 585 m for a female. Basic field census did not show significant difference between sexes for the median distance moved (Wilcoxon rank-sum test, Females: n = 106, median = 49 m; Males: n = 295, median = 30 m, W = 12430.5, p = 0.1346).

### Capture-mark-recapture multistate models

**Figure 2:**
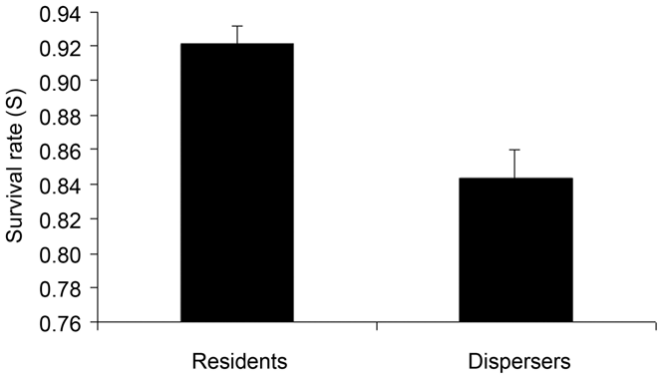
Mean (± SE) daily survival of residents and dispersers estimated from the best model {S(state)p(t+sex)psi(sex+sr+d)}.

**Figure 3:**
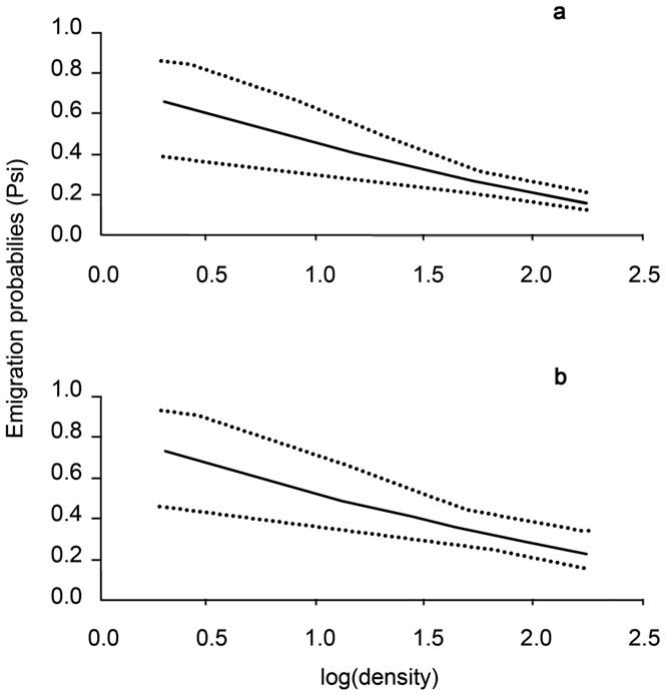
Emigration probabilities (i.e. from the starting section to a different section of arrival) against the density for males (a) and females (b). The dashed lines correspond to the confidence interval.

**Figure 4:**
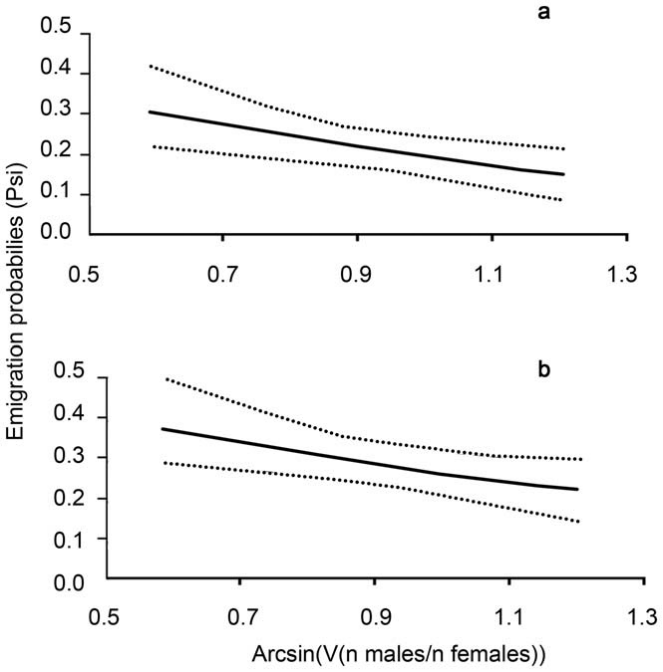
Emigration probabilities (i.e. from the starting section to a different section of arrival) against the sex-ratio for males (a) and females (b). The dashed lines correspond to the confidence interval.

The best models are shown in the Table 2. The most parsimonious model without covariates was {S(state)p(t+sex)psi(sex)} (model 17 in Table 2); which considered an effect of dispersal status (state) on survival probability, of time (t) and sex on recapture probability, and of sex on dispersal propensity. The likelihood ratio tests conducted between the 5 competing best models (models 1–5, Table 2) revealed that the most parsimonious one, which included covariates, was {S(state)p(t+sex)psi(sex+sex-ratio+density)} (model 3 in Tables 2 and 3). Daily survival depended on state and was 0.843±0.018 (mean ± SE) for dispersers and 0.921±0.010 for residents (Fig. 2). Recapture probability varied with time but in the same way for both sexes. Finally, emigration probability depended on sex as females were more likely to move than males (psi females = 0.280±0.035; psi males = 0.230±0.027). In addition, only density and sex-ratio of the starting section negatively influenced emigration probability for both sexes (Fig. 3 and 4). Wing length did not affect survival or dispersal as models with this covariate had no statistical support (ΔAICc≫10 and AICc weights = 0).

**Table 2:** Model selection for survival (S), recapture (p) and emigration (psi) probabilities.

Model	K	AICc	ΔAICc	AICc Weights	Model Likelihood	Deviance
1 {S(state+sr+d)p(t+sex)psi(sex+sr+d)}	22	9704.53	0	0,199	1	9659.86
2 {S(state+sr)p(t+sex)psi(sex+sr+d)}	21	9705.04	0.512	0,154	0.774	9662.44
**3 {S(state)p(t+sex)psi(sex+sr+d)}**	**20**	**9705.30**	**0.775**	**0,135**	**0.679**	**9664.75**
4 {S(state+d+sr)p(t+sex)psi(sex*sr+d)}	23	9705.43	0.901	0,127	0.637	9658.70
5 {S(state+d)p(t+sex)psi(sex+sr+d)}	21	9706.08	1.550	0,092	0.461	9663.47
6 {S(state+d+sr)p(t+sex)psi(sex*d+sr)}	23	9706.59	2.061	0,071	0.357	9659.86
7 {S(state+sr+d)p(t+sex)psi(sex+d)}	21	9707.15	2.624	0,054	0.269	9664.55
8 {S(state+d+sr)p(t+sex)psi(sex*(sr+d))}	24	9707.49	2.964	0,045	0.227	9658.70
9 {S(state+sr)p(t+sex)psi(sex*(sr+d))}	23	9707.95	3.421	0,036	0.181	9661.23
10 {S(state*sr+d)p(t+sex)psi(sex*(sr+d))}	25	9708.90	4.374	0,022	0.112	9658.05
11 {S(state+d)p(t+sex)psi(sex*(sr+d))}	23	9709.04	4.508	0,021	0.105	9662.31
12 {S(state*d+sr)p(t+sex)psi(sex*(sr+d))}	25	9709.45	4.919	0,017	0.086	9658.59
13 {S(state*(sr+d))p(t+sex)psi(sex*(sr+d))}	26	9710.69	6.158	0,009	0.046	9657.76
14 {S(state)p(t+sex)psi(sex*d)}	20	9710.89	6.366	0,008	0.042	9670.34
15 {S(state+sr+d)p(t+sex)psi(sex+sr)}	21	9711.90	7.370	0,005	0.025	9669.29
16 {S(state+sr)p(t+sex)psi(sex+sr)}	20	9714.06	9.538	0,002	0.009	9673.52
17 {S(state)p(t+sex)psi(sex)}	18	9715.29	10.763	0,001	0.005	9678.84
18 {S(state)p(t+sex)psi(sex*sr)}	20	9716.33	11.802	0,001	0.003	9675.78
19 {S(state+sex)p(t+sex)psi(sex)}	19	9716.90	12.376	0	0.002	9678.41
20 {S(state)p(t+sex)psi(.)}	17	9718.48	13.949	0	0.001	9684.08

Factors: state (resident or disperser), t = time (day), and sex (male or female).

Covariates: sr = sex-ratio and d = density.

K = number of parameters.

Competing best models, having ΔAICc values lower than 2, are models 1 to 5 and the one including all significant effects is model 3 in bold (see Table 3 for LRT statistics associated). The 20 best models are shown.

**Table 3:** Results of the Likelihood Ratio Tests (LRT) to evaluate which model, between the five best models (ΔAICc<2, see Table 2), includes all significant factors.

Models compared	tested effect (parameter)	Chi2	d.f.	p value
**1** vs 4	sex.sr (p)	1.160	1	0.2815
1 vs **2**	d (S)	2.570	1	0.1089
1 vs **5**	sr (S)	3.608	1	0.0575
2 vs **3**	sr (S)	2.318	1	0.1279
**3** vs 5	d (S)	1.280	1	0.2579

The best model for each pairwise comparison is shown in bold and corresponds to the simplest model (p>0.05). Overall, the model including all significant effects is the model 3, {S(state)p(t+sex)psi(sex+sr+d)}, as model 3 is better than models 2 and 5, which are better than model 1, which is better than model 4.

## Discussion

We defined here dispersal as movements leaving individuals outside of their home ranges. Results showed that this definition is biologically sounded because leaving the home range (i.e. moving more than 50 m) is a decision that will entail survival cost, with survival of dispersing individuals being 8% less than survival of residents. We also found evidence of negatively density-dependent dispersal. Finally, we showed here that dispersal was depending on sex but not on wing length.

We pointed out that females dispersed more than males (i.e. they left their section of origin more frequently than males did) but had not necessarily a larger dispersal distance (i.e. distance covered during the study). Most of CMR studies only compare dispersal distances between sexes, which may lead to erroneous results regarding differential rates of dispersal among sexes. Hence we think that estimating the emigration probability from individual home-range can be more efficient to show a sex-biased dispersal and dissociate routine movements from dispersal. Wing length did not seem to account for dispersal ability in our study: we did not find evidence that larger individuals dispersed farther or survived better contrary to other studies on insects [8], [9], [10], [36], [67], [68]. However, such a relationship between body size and mobility was not observed in damselfly and butterfly species [69], [70], [71].

We found a negative relationship between the probability to move and population density in *C. splendens* (see also [40], [48]). This is in line with the use of conspecific as proxy of habitat quality in a weakly spatially autocorrelated environment. Under such conditions, negative density-dependent dispersal is the best solution to achieve a fine tuning of distribution of individuals according to their fitness expectation. Furthermore, individuals moved less when the sex-ratio was male biased. This result is congruent with the lek mating system observed in another calopterid species where males aggregate waiting for females [39]. Females were also found in patches with high male density where opportunity for mate choice might be better. Consequently, female-biased dispersal we recorded here might be due to the avoidance of male harassment in high density patches [47], and corresponds to the hypothesis of a lek mating systems in Calopterygidae [39].

The lower survival observed in dispersers suggests a dispersal cost. To our knowledge this is the first time that a CMR study shows such high survival differences between residents and dispersers. However, we only have access to survival of successful dispersers (i.e. recaptured on their arrival site), then we do not have the survival cost during transfer. But, this cost would even decrease survival estimations of dispersers rather than the reverse. Thus, our results are conservative in that sense and might represent a minimal survival cost. Survival costs can be due to predation by spiders, birds or fishes [72], [73], [74]. A predation cost implicates that residential individuals are less active and as such less trapped in spider webs or less conspicuous to bird predators than dispersers. Survival cost can also be the outcome of invested energy in flight. Nonetheless, we cannot rule out the hypothesis of permanent emigration. Permanent emigration corresponds to individuals leaving the study site which might bias survival estimate [75]. We tried to limit this bias avoiding a boundary effect and knowing ‘ecological neighbourhood’ [76]. A boundary effect means that individuals closed to the start or the end of the transect are more likely to leave the study site. Here, we chose our study system in such a way that our transect was isolated on both side by 700 m of unsuitable habitats. Given the high spatial fidelity of males and females (median displacements of 30 m and 49 m respectively), we think that this boundary effect is marginal. Moreover, negative density-dependent dispersal should also contribute to reduce emigration out of the study site.

Freshwater insects are often considered to form metapopulations [77], [78]. Our results suggest that density dependent dispersal and the lek mating system could be related: individuals in a lek are more abundant, and hence the lek is more attractive, which means that individuals from low density sites are attracted to high density sites (i.e. dispersal is negatively density-dependent). Accordingly, the ultimate driver of the spatial structure of *C. splendens* could be the evolutionary interplay between the lek mating system and density-dependent dispersal.
